# Plasmodium falciparum Apicomplexan-Specific Glucosamine-6-Phosphate *N*-Acetyltransferase Is Key for Amino Sugar Metabolism and Asexual Blood Stage Development

**DOI:** 10.1128/mBio.02045-20

**Published:** 2020-10-20

**Authors:** Jordi Chi, Marta Cova, Matilde de las Rivas, Ana Medina, Rafael Junqueira Borges, Pablo Leivar, Antoni Planas, Isabel Usón, Ramón Hurtado-Guerrero, Luis Izquierdo

**Affiliations:** a ISGlobal, HospitalClinic–Universitat de Barcelona, Barcelona, Spain; b Institute of Biocomputation and Physics of Complex Systems (BIFI), University of Zaragoza, Zaragoza, Spain; c Crystallographic Methods, Institute of Molecular Biology of Barcelona (IBMB–CSIC), Barcelona, Spain; d Departamento de Física e Biofísica, Instituto de Biociências, Universidade Estadual Paulista (UNESP), Botucatu, Brazil; e Laboratory of Biochemistry, Institut Químic de Sarrià, Universitat Ramon Llull, Barcelona, Spain; f ICREA, Institució Catalana de Recerca i Estudis Avançats, Barcelona, Spain; g Copenhagen Center for Glycomics, Department of Cellular and Molecular Medicine, School of Dentistry, University of Copenhagen, Copenhagen, Denmark; h Laboratorio de Microscopías Avanzada (LMA), University of Zaragoza, Zaragoza, Spain; i Fundación ARAID, Zaragoza, Spain; University of Geneva

**Keywords:** malaria, metabolism, *Plasmodium falciparum*, UDP-*N*-acetylglucosamine, aminosugar pathway, apicomplexan parasites

## Abstract

UDP-*N*-acetylglucosamine (UDP-GlcNAc), the main product of the hexosamine biosynthetic pathway, is an important metabolite in protozoan parasites since its sugar moiety is incorporated into glycosylphosphatidylinositol (GPI) glycolipids and *N*- and *O*-linked glycans. Apicomplexan parasites have a hexosamine pathway comparable to other eukaryotic organisms, with the exception of the glucosamine-phosphate *N*-acetyltransferase (GNA1) enzymatic step that has an independent evolutionary origin and significant differences from nonapicomplexan GNA1s. By using conditional genetic engineering, we demonstrate the requirement of GNA1 for the generation of a pool of UDP-GlcNAc and for the development of intraerythrocytic asexual Plasmodium falciparum parasites. Furthermore, we present the 1.95 Å resolution structure of the GNA1 ortholog from Cryptosporidium parvum, an apicomplexan parasite which is a leading cause of diarrhea in developing countries, as a surrogate for P. falciparum GNA1. The in-depth analysis of the crystal shows the presence of specific residues relevant for GNA1 enzymatic activity that are further investigated by the creation of site-specific mutants. The experiments reveal distinct features in apicomplexan GNA1 enzymes that could be exploitable for the generation of selective inhibitors against these parasites, by targeting the hexosamine pathway. This work underscores the potential of apicomplexan GNA1 as a drug target against malaria.

## Abstract (precis)

Apicomplexan parasites cause a major burden on global health and economy. The absence of treatments, the emergence of resistances against available therapies, and the parasite’s ability to manipulate host cells and evade immune systems highlight the urgent need to characterize new drug targets to treat infections caused by these parasites. We demonstrate that glucosamine-6-phosphate *N*-acetyltransferase (GNA1), required for the biosynthesis of UDP-*N*-acetylglucosamine (UDP-GlcNAc), is essential for P. falciparum asexual blood stage development and that the disruption of the gene encoding this enzyme quickly causes the death of the parasite within a life cycle. The high-resolution crystal structure of the GNA1 ortholog from the apicomplexan parasite C. parvum, used here as a surrogate, highlights significant differences from human GNA1. These divergences can be exploited for the design of specific inhibitors against the malaria parasite.

## INTRODUCTION

In 2018 there were more than 200 million clinical cases of malaria, a parasitic disease caused by species of the genus *Plasmodium*, that led to more than 400,000 deaths. Most of them were caused by Plasmodium falciparum parasites in children under 5 years old in Sub-Saharan Africa (1). Despite the strong calls for malaria elimination and the important advances made in this direction since the beginning of the 21st century, recent reports indicate the lack of significant progress in reducing global malaria cases in the last few years (1, 2). This slowdown, together with the recent emergence of resistance to first-line antimalarial treatments (3, 4), poses an imminent threat that endangers the outstanding improvements against the disease made in the last decades. Therefore, new approaches to control malaria, including the characterization of essential metabolic pathways and drug targets and the development of new chemotherapeutic treatments, are urgently needed (5, 6).

Malaria infection begins with the inoculation of P. falciparum sporozoites into the human dermis through the bite of a parasite-infected female *Anopheles* mosquito. Sporozoites enter the bloodstream and reach the liver, where they invade and develop inside hepatocytes, giving rise to exoerythrocytic merozoites. Released merozoites then invade red blood cells (RBCs) and initiate multiple ∼48-h rounds of asexual replication, leading to an exponential parasite growth which is responsible for disease symptoms (7). A small fraction of blood stage parasites, about 1 to 2%, differentiate into sexual forms and produce male and female gametocytes, transmissible to *Anopheles* mosquitoes during a blood meal. Once inside the mosquito midgut, gametocytes form male and female gametes that undergo sexual recombination to produce ookinetes and then oocysts, which end up releasing new sporozoites. Therefore, antimalarial drugs should not only eliminate asexual blood stage parasites, but also target preerythrocytic and sexual stages to prevent and block disease transmission, contributing to malaria elimination strategies (4, 8).

Recent works highlight the importance of the amino sugar pathway and its final product, the sugar nucleotide UDP-*N*-acetylglucosamine (UDP-GlcNAc), for the survival of human and murine malaria parasites along different life stages (9–12). Sugar nucleotides are activated sugar precursors used by glycosyltransferases to produce glycoconjugates, and UDP-GlcNAc is required for the biosynthesis of glycosylphosphatidylinositol (GPI) anchors and *N*-glycans (13). These glycoconjugates play essential roles in P. falciparum (14, 15) and other protozoan parasites (16, 17). Malaria parasites present a conventional amino sugar metabolic route for the synthesis of UDP-GlcNAc (18) with the particularity that the glucosamine-6-phosphate *N*-acetyltransferase (GNA1) enzyme belongs to an apicomplexan-specific gene family with unique sequence features and conserved motifs (10). These specificities might render GNA1 amenable for selective inhibition. In this work, we demonstrate the importance of the enzyme for the biosynthesis of UDP-GlcNAc and the survival of the parasite along its intraerythrocytic life cycle, outlining the distinctive features of apicomplexan GNA1s by reporting the crystal structure of the C. parvum homologous GNA1 enzyme.

## RESULTS

### Conditional disruption of the *gna1* gene in P. falciparum.

**FIG 1:**
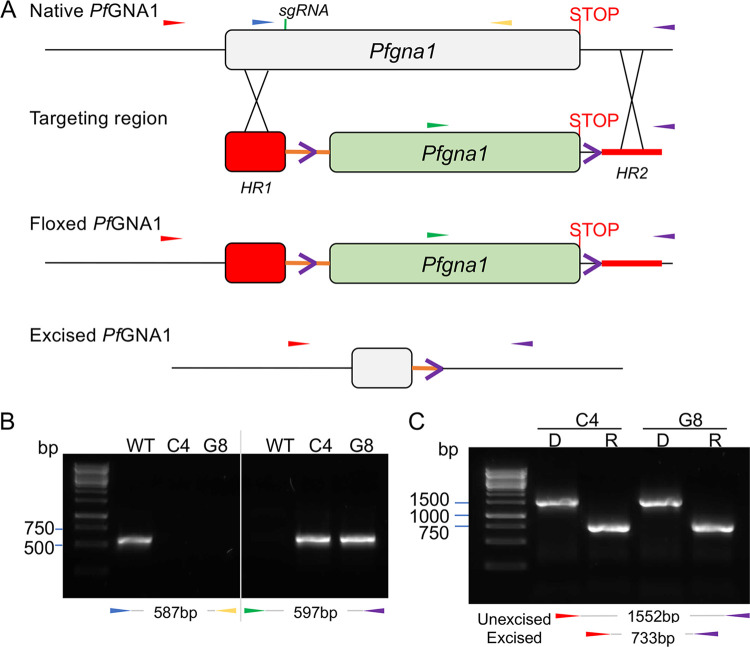
Conditional disruption of P. falciparum *gna1* gene. (A) P. falciparum *gna1* is a single-exon gene. Using Cas9-assisted genome editing, 696 bp of the native *gna1* open reading frame were replaced with a *loxP* (purple open arrowhead)-containing intron (orange line), and an additional *loxP* site that flanks a recodonized version of P. falciparum *gna1*. Integration of this sequence by double crossover homologous recombination was promoted by (i) Cas9 endonuclease-mediated double-strand DNA breaks, guided by a single guide RNA (sgRNA) targeting a 20-nucleotide region of *Pfgna1* (green arrows); and (ii) the addition of homologous sequences (red box at the 5′ region and thick red line at the 3′ region) to either side of the *loxP* sites. Closed colored arrows indicate the primer binding sites. Rapamycin-induced site-specific recombination between the *loxP* sites removes the recodonized part of the gene (green box) encompassing 223 amino acids of the 291 amino acids of the *Pf*GNA1 enzyme. (B) PCR analysis of *gna1-loxP* clones C4 and G8 confirms successful gene editing. Genomic DNA from parental 3D7 (WT) parasites or C4 and G8 clones was used as the template for PCR using the primers indicated in panel A. The numbers between the colored arrows indicate the expected amplicon size. (C) Truncation of the rapamycin-induced *gna1* gene. *gna1-loxP* C4 and G8 clones were analyzed by PCR ∼29 h after treatment with DMSO (D) or rapamycin (R) using the primers identified in panel A. Excision reduces the amplicon from 1,552 bp to 733 bp, disrupting *gna1*.

Previous work showed the P. falciparum *gna1* gene could not be ablated but its coding sequence could be engineered to include synonymous substitutions, strongly suggesting an indispensable role for asexual intraerythrocytic parasites (10). Thus, to gain further insights into the importance of the gene for P. falciparum asexual blood stage development, we engineered a *gna1* conditional null mutant using a rapamycin-inducible dimerizable Cre-recombinase (DiCre) system (19). By CRISPR-Cas9 technology (20), we introduced two *loxP* sites in the gene, one in a synthetic intron (21) within the *gna1* open reading frame, and another in the 3′ untranslated region (Fig. 1A). DiCre-mediated excision of the floxed sequence could be induced by adding rapamycin into the media. The ablation disrupts a region comprising 669 bp of the *gna1* gene, including the Gcn5-related *N*-acetyltransferase (GNAT) domain, conserved in enzyme families that use acetyl-CoA to acetylate substrates (22, 23). The genomic modification was engineered in the DiCre-expressing P. falciparum line 3D7 II.3 (19).

Confirmation of the appropriate modification of the *gna1* gene after transfection was assessed by diagnostic PCR. Subsequent limiting dilution cloning of the *gna1* locus-modified parasites resulted in the isolation of *gna1*-*loxP*-C4 and *gna1*-*loxP*-G8 clones, in which the modification of the locus was reconfirmed again by PCR (Fig. 1B). Efficiency of the conditional excision of the floxed *gna1*-*loxP* clones was assessed by the addition of rapamycin or dimethyl sulfoxide (DMSO; vehicle control) in ring stage synchronized cultures. Cells were treated for 1 h, followed by washing and incubation for 29 h to allow parasite maturation. Genomic DNA from the clones was used for diagnostic PCR using primers annealing in the homology regions that demonstrated the efficient excision of *gna1-loxP* (Fig. 1C). Both clones were used in all subsequent experiments.

### Disruption of the *gna1* gene leads to the inhibition of parasite growth and reduction of UDP-GlcNAc pools.

**FIG 2:**
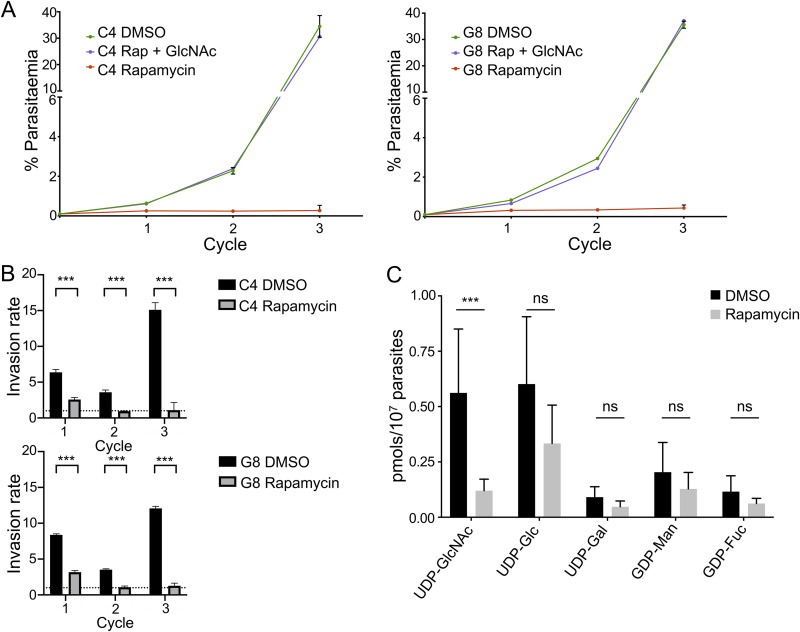
P. falciparum *gna1* disruption produces developmental arrest and reduces the UDP-GlcNAc pool. (A) Synchronous ring-stage *gna1-loxP* growth curves of C4 and G8 clones showing parasite replication over the course of three erythrocytic cycles when grown in the presence of DMSO, rapamycin, or rapamycin + 10 mM GlcNAc. Data were averaged from three biological replicates and presented as the mean ± standard deviation (SD). (B) Invasion rates of DMSO- or rapamycin-treated cultures (clones C4 and G8). The graphs show mean ± SD values of three replicates and significance was assessed using unpaired *t* tests. (C) Quantification of UDP-GlcNAc and other sugar nucleotide pools in late stages (mature trophozoites) of *gna1-loxP* parasites treated with rapamycin. Values indicate the sugar nucleotide concentration ± SD of six different biological replicates. Unpaired *t* tests were used to compare differences; ***, *P* < 0.05; ns, not significant.

GNA1 catalyzes the acetylation of glucosamine-6-phosphate (GlcN6P) to generate *N*-acetylglucosamine-6-phosphate (GlcNAc6P), which is needed for the biosynthesis of UDP-GlcNAc, a required precursor for both GPI-anchors and *N*-glycans (10). These glycoconjugates are critical for parasite viability and, hence, a defect in their synthesis may largely affect P. falciparum development. To ascertain the effect of *gna1* loss in asexual parasites, rapamycin-treated cells were grown and monitored by flow cytometry over ∼144 h (i.e., three intraerythrocytic developmental cycles [IDCs]). Upon rapamycin-induced *gna1-loxP* excision, parasites failed to expand over three IDCs, whereas DMSO-treated parasites grew normally. Neither rapamycin nor DMSO affect parasite growth at the incubation times and concentrations used (19). Interestingly, parasite growth in rapamycin-treated cultures was rescued by supplementing the medium with 10 mM GlcNAc, suggesting the presence of a metabolic pathway for the uptake and activation of free GlcNAc (Fig. 2A). Rapamycin-treated cultures showed an ∼2.5-fold invasion rate reduction in the first IDC, indicating the expansion of a small proportion of viable parasites that likely reflects the kinetics of DiCre-mediated excision (19), GNA1 turnover, and/or the dynamics of sugar nucleotide pools (24) (Fig. 2B). Nevertheless, invasion rates were reduced to 1 (i.e., absence of growth) in rapamycin-treated cultures at cycles 2 and 3. Parasite morphology along the IDCs was normal in DMSO treated-cultures, gradually losing synchronization after 2 to 3 cycles. In contrast, in rapamycin-treated cultures, growth-arrested parasites accumulated as trophozoites and schizonts, indicating that *gna1* is needed for parasite development through mature stages (Fig. S1 in the supplemental material). To precisely assess the effect of *Pf*GNA1 loss on UDP-GlcNAc levels, we measured and compared the sugar nucleotide dynamic pools of DMSO-treated parasites with those of rapamycin-treated parasites. Upon rapamycin treatment, the UDP-GlcNAc pool was significantly reduced, despite that levels of other sugar nucleotides remained similar (Fig. 2C). Therefore, without discarding potential *Pf*GNA1 moonlighting activities essential for parasite development, the results strongly suggest that *gna1* disruption prevents GlcN6P acetylation by *Pf*GNA1 and reduces the pool of UDP-GlcNAc, hindering P. falciparum asexual intraerythrocytic growth.

### C. parvum GNA1 structural analysis.

**FIG 3:**
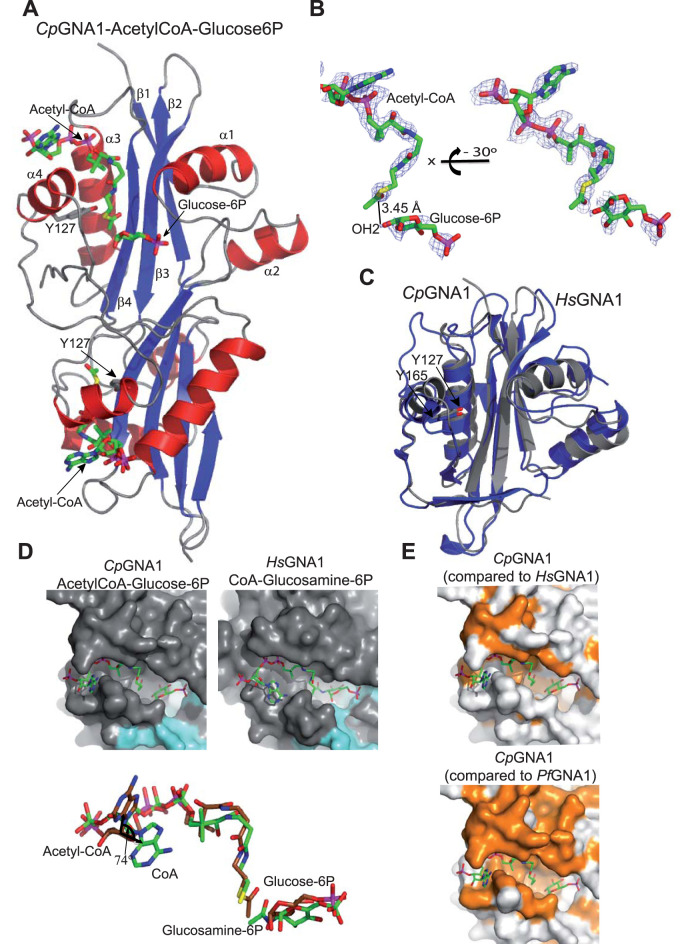
*Cp*GNA1 and human *Hs*GNA1 folding and conservation of the substrate-binding site. (A) Overall crystal structure of *Cp*GNA1. Secondary structure is shown in blue (strands) and red (helices) for both monomers. The highly conserved active site tyrosine residue is shown as sticks with gray carbon atoms. Acetyl-CoA and Glc6P are shown as sticks with green carbon atoms. (B) Two different views of the electron density map F_O_ F_C_ (blue), which is contoured at 2.0 σ for acetyl-CoA and glucose-6P. Note that the electron density is of good and moderate quality for acetyl-CoA and glucose-6P, respectively. (C) Superposition of the monomers from *Cp*GNA1 (gray) and *Hs*GNA1 (blue). The equivalent Tyr127 of *Cp*GNA1 is Tyr165 in the *Hs*GNA1 (shown as blue carbon atoms). (D) Surface representation (upper panel) of *Cp*GNA1 and *Hs*GNA1 structures colored in gray. Note the different shapes of the binding site between both enzymes and the differences of acetyl-CoA and CoA, which are mostly evidenced by large conformational changes between the adenine moieties (see the lower panel). (E) Surface representation of *Cp*GNA1 in which the degree of conservation of amino acid residues surrounding the binding site are color coded. *Cp*GNA1 was compared with *Hs*GNA1 (upper panel) and *Pf*GNA1 (lower panel). Note that no models were generated for either *Hs*GNA1 or *Pf*GNA1 and that the differences were exclusively based on amino acid sequence alignments. Orange and white represent identity/conservative substitutions and nonconservative substitutions, respectively. The ligands are represented as sticks with green carbon atoms, except for acetyl-CoA and glucose-6P in lower panel of (D), in which they are colored as brown carbon atoms.

*Pf*GNA1 activity is crucial for the development of the malaria parasite and, therefore, understanding the structural differences with human GNA1 (*Hs*GNA1) may contribute to the discovery of *Pf*GNA1-selective inhibitors. To further characterize the enzyme and, given our inability to express and purify sufficient amounts of active *Pf*GNA1 (10), we used *Cp*GNA1 as a surrogate, considering that it belongs to the same Apicomplexa-specific GNA1 family and can be purified fairly well with a high yield (10). The percentage of pairwise identity within the ca. 90-amino acid conserved region of the *Cp*GNA1 and *Pf*GNA1 GNAT domains is 48.8% (similarity 61.9%). The first *Cp*GNA1 structure, determined from crystals belonging to the space group P2_1_2_1_2, with one monomer in the asymmetric unit, was solved by using the multisolution parallel phasing software ARCIMBOLDO (25). Extension of the placed fragments with SEQUENCE SLIDER (26) was required. The dimeric structure of *Cp*GNA1 in complex with acetyl-CoA and glucose-6-phosphate (Glc6P) from data at 1.95 Å in space group P2_1_2_1_2_1_ was solved by molecular replacement with Phaser (27) using the monomeric structure (Fig. 3A and B), and further assessing ligand binding by representing unbiased sim-omit maps (Fig. S2). Note that Glc6P was used previously as a pseudosubstrate to get insights into GNA1-GlcN6P recognition and catalysis (Fig. 3A and B) (28). *Cp*GNA1 is found as a dimer, as is the case for other reported GNA1 crystal structures (29–31). *Cp*GNA1 adopts the typical GCN5-related *N*-acetyltransferase (GNAT) fold previously described in GNA1 enzymes, though with some variations (see below; Fig. 3A) (29). The *Cp*GNA1 structure consists of a central twisted four-stranded antiparallel β-sheet surrounded by four α helices (Fig. 3A). This feature is conserved with *Hs*GNA1, though this enzyme also contains additional β-strands and α-helices (Fig. 3C and Fig. S3) (32). The superposition of *Cp*GNA1 and *Hs*GNA1 monomers gives a root mean square deviation (RMSD) of 1.68 Å on 132 C*α* atoms, suggesting some conformational differences (Fig. 3C). Additionally, *Hs*GNA1 resembles the more typical signature of the GNAT superfamily than does *Cp*GNA1, supporting our previous data that suggest an independent evolutionary origin and highlight the presence of significant differences between apicomplexan and nonapicomplexan GNA1s, as discussed in reference 10. Actually, the Apicomplexa GNA1 family shows a taxonomic distribution restricted to apicomplexan organisms (10). In agreement, the typical topology for GNAT-fold-adopting members (β1-α1-α2-β2-β3-β4-α3-β5-α4-β6-β7) (32–34) is more consistent with that found for *Hs*GNA1 (α1-β1-α2-α3-β2-β3-α4-β4-α5-β5-α6-β6) and more distant to that found for *Cp*GNA1 (β1-α1-α2-β2-β3-β4-α3-α4), which contains significantly fewer secondary structure elements (Fig. S3). Remarkably, the highly conserved tyrosine of the active site, also present in *Cp*GNA1 (Tyr127), establishes a hydrogen bond between its hydroxyl group and the thioester sulfur, leading to an increase in the electrophilic character of the carbonyl group of the acetyl-CoA molecule during catalysis (28, 30) (Fig. 3A). This is key for favoring a direct nucleophilic attack by the GlcN6P amine (28) that promotes catalysis. Note the proximity of the Glc6P OH_2_ that mimics the amine group of GlcN6P to the carbonyl group of acetyl-CoA (distance of 3.45 Å; Fig. 3B), which clearly supports that the same mechanism might take place for *Cp*GNA1.

### *Cp*GNA1 active site.

**FIG 4:**
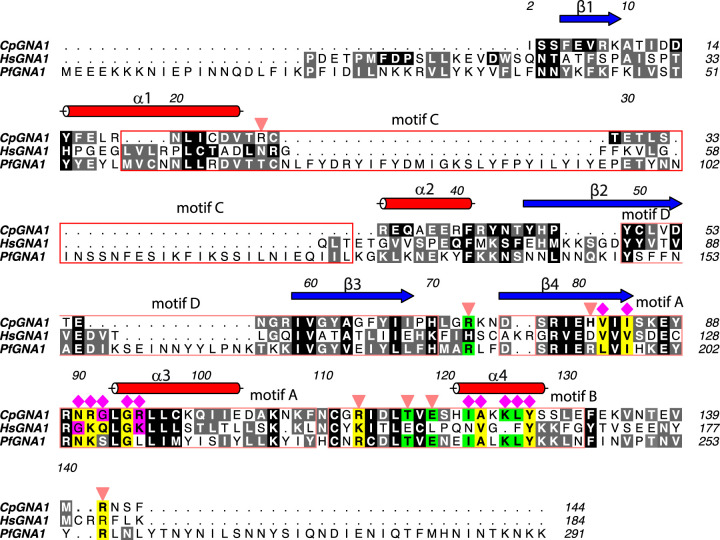
Multiple sequence alignment of *Cp*GNA1, *Hs*GNA1, and *Pf*GNA1. The *Cp*GNA1 residues that interact with acetyl-CoA and Glc6P and the grade of conservation with other *Cp*GNA1 orthologs based on these interactions are highlighted as follows: yellow (full conservation); green (conserved residues between *Cp*GNA1 and *Pf*GNA1); magenta (conserved residues between *Cp*GNA1 and the *Hs*GNA1); and black/gray colors that indicate the degree of conservation between the three orthologs. Residues interacting with acetyl-CoA and Glc6P are also displayed as magenta diamonds and inverted orange triangles, respectively. Shown above the sequence for *Cp*GNA1, in red and blue, is displayed the secondary structure elements (α-helices and β-strands, respectively). Note that residues 90 to 93 of *Cp*GNA1 align well at the structural level with the equivalent residues in *Hs*GNA1 but not at the sequence level. This is evidenced for *Cp*GNA1 Asn90 and *Hs*GNA1 Gln132, and *Cp*GNA1 Gly92 and *Hs*GNA1 Gly130. The four different motifs present in the GNAT family of higher eukaryotes are represented as open red boxes (32). The secondary structures are mostly conserved between *Cp*GNA1 and *Hs*GNA1 in motifs D and A, and to a lesser extent in motif B (see Fig. S3 for a comparison of the secondary structure elements between both enzymes), exemplifying the large differences between the enzymes from higher eukaryotes and the Apicomplexa phylum.

**FIG 5:**
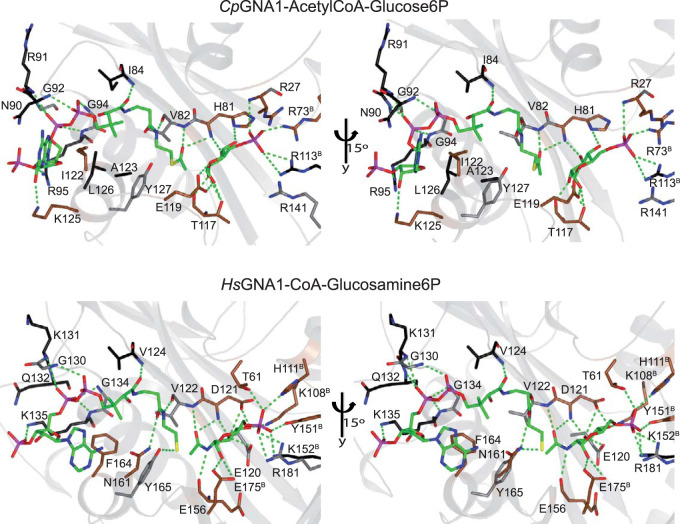
Comparison of the *Cp*GNA1 and human *Hs*GNA1 active sites. Two different views are depicted for each active site to better visualize the interactions between the ligands and the amino acids. The protein backbone is shown as a gray ribbon. Ligands are shown as sticks with green carbon atoms. *Hs*GNA1 residues interacting with the ligands are shown for comparison and illustrative purposes but will not be discussed in detail. Protein–ligand hydrogen bonds are shown as dotted green lines. Identical, homologous, and dissimilar residues between both enzymes are colored in gray, black, and brown carbon atoms, respectively. Note that the superscript B in several residues defines residues that come from chain B.

A close inspection of the active site of *Cp*GNA1 and its comparison with the *Hs*GNA1 active site showed that both the GlcN6P and the acetyl-CoA binding sites have different shapes (Fig. 3D), and are also different at the sequence level (Fig. 3E and Fig. 4). This is more evident for the acetyl-CoA binding site, in which significant differences in the acetyl-CoA/CoA phosphorylate ADP moiety are found. For example, the angle between both adenine moieties is close to perpendicular (74°) and the distance between the free amines is large (5.4 Å) (Fig. 3D). Note that the angle is formed between the C1B of the phosphorylated ribose and the two C4A of the adenine moieties. However, the *Cp*GNA1 active site and the predicted *Pf*GNA1 active site are more similar (Fig. 3B), as anticipated in our previous work (10). There are 19 residues of *Cp*GNA1 engaged in the recognition of acetyl-CoA and Glc6P (Fig. 4 and Fig. 5). Some of these residues forming the GlcN6P binding site come from both monomers (see Fig. 5). Out of these 19 residues, 11 residues are conserved between *Hs*GNA1 and *Cp*GNA1, whereas 15 residues are conserved between *Cp*GNA1 and *Pf*GNA1. Two residues are unique in *Cp*GNA1, though these residues interact with the ligands through their backbones (Fig. 4 and Fig. 5).

The adenine moiety and the dimethyl group of acetyl-CoA establish CH-π and CH-CH interactions with Ile122 and Ala123/Leu126, respectively. The phosphorylated ribose, pyrophosphate, pantothenic acid, the β-mercaptoethylamine, and the acetyl moieties are tethered via hydrogen bonds to the Arg95 side chain and Lys125 backbone, Asn90/Arg91/Gly92/Gly94/Arg95 backbones, Ile84 backbone, Val82 backbone, and His81/Val82 backbones, respectively (Fig. 5). The Glc6P is tethered via hydrogen bonds to the His81/Thr117/Arg27 backbones and Arg73^B^/His81/Arg113^B^/Thr117 side chains (Fig. 5). Note that the sugar moiety is also recognized by residues belonging to the chain B in both *Cp*GNA1 and *Hs*GNA1 (Fig. 5).

### C. parvum GNA1 site-directed mutagenesis.

Based on the structural information, nine *Cp*GNA1 site-specific mutants were created to study the contribution of specific residues to *Cp*GNA1 binding and activity. All mutants were designed to disrupt the binding of either acetyl-CoA (N90A, I122G, and Y127A) or GlcN6P (R73A, R141A, R113A, T117A, E119A, and H81A) (Fig. 5). Note that in our structure, the critical Tyr127 and Arg141 were very close to the ligands but did not show any interaction (Fig. 5). However, and considering the importance of the Tyr counterparts in other orthologs (28), it would be expected that the mutation of Tyr127 to Ala would diminish or abolish the activity, as shown below. The selected amino acids were replaced by alanine (A) in all cases except for Ile122, for which isoleucine was replaced by glycine (I122G).

**FIG 6:**
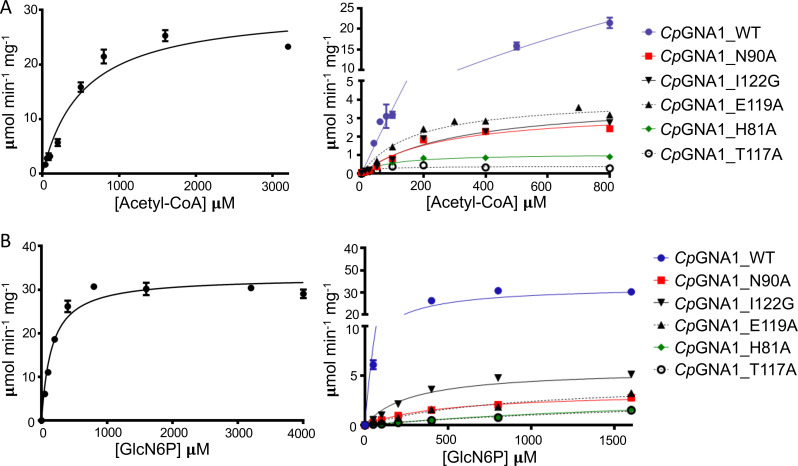
*Cp*GNA1 kinetics. (A) *Cp*GNA1 steady-state velocities for recombinant WT *Cp*GNA1 (left panel) and *Cp*GNA1 mutants (right panel) using different concentrations of acetyl-CoA at a fixed (2,000 μM) concentration of GlcN6P. (B) *Cp*GNA1 steady-state velocities for recombinant WT *Cp*GNA1 (left panel) and *Cp*GNA1 mutants (right panel) using different concentrations of GlcN6P at a fixed (500 μM) concentration of acetyl-CoA.

The steady-state kinetic parameters of wild type (WT) and *Cp*GNA1 mutants were studied by an *in vitro* colorimetric assay by monitoring CoA release, with increasing concentrations of acetyl-CoA at a fixed concentration of GlcN6P and vice versa (Fig. 6). The R73A, R113A, R141A, and Y127A activities were almost negligible, hampering the calculation of reliable kinetic parameters (Table 1).

**TABLE 1:** Comparison of the kinetic parameters between WT and mutant *Cp*GNA1

Enzyme	*V*_max_ (μmol min^−1^ μg^−1^)	*k*_cat_ (s^−1^)^*a*^	Acetyl-CoA		GlcN-6P	
			*K*_m_ (μM)	*k*_cat_/*K*_m_ (s^−1^ μM^−1^)	*K*_m_ (μM)	*k*_cat_/*K*_m_ (s^−1^ μM^−1^)
*Cp*GNA1-WT	32.86 ± 0.78	10.31	531 ± 80.33	1.94 × 10E-2	153.9 ± 16.46	6.7 × 10E-2
*Cp*GNA1-N90A	3.53 ± 0.06	1.10	252.4 ± 44.43	4.37 × 10E-3	532.7 ± 24.84	2.07 × 10E-3
*Cp*GNA1-E119A	4.09 ± 0.19	1.28	170.4 ± 25.06	7.5 × 10E-3	1136 ± 125.2	1.13 × 10E-3
*Cp*GNA1-I122G	3.99 ± 0.34	1.25	309.2 ± 54.94	4.03 × 10E-3	335.7 ± 33.78	3.71 × 10E-3
*Cp*GNA1-H81A	1.07 ± 0.04	0.34	88.9 ± 10.98	3.77 × 10E-3	4007 ± 1425	8.36 × 10E-5
*Cp*GNA1-T117A	0.4 ± 0.03	0.12	33.41 ± 11.82	3.73 × 10E-3	3245 ± 346.4	3.84 × 10E-5
*Cp*GNA1-R113A	ND	ND	ND	ND	ND	ND
*Cp*GNA1-R73A	ND	ND	ND	ND	ND	ND
*Cp*GNA1-R141A	ND	ND	ND	ND	ND	ND
*Cp*GNA1-Y127A	ND	ND	ND	ND	ND	ND

a *k*_cat_ values were calculated using the molecular weight of the protein itself; the GST tag was not considered for the calculations since it does not affect *Cp*GNA1 activity. ND, not determined.

*Cp*GNA1 mutant kinetics data showed a general decrease in catalytic efficiency (*k*_cat_/*K*_m_) and enzyme maximal specific activity (defined here as *V*_max_) (Table 1). The catalytic efficiency of *Cp*GNA1 mutants decreased between 2.6- and 5.2-fold and 18.6- to 1,754-fold for acetyl-CoA and GlcN6P, respectively. *V*_max_ also decreased between 8- and 83-fold, depending on the mutant enzyme. With respect to the *K*_m_ values, all mutants displayed lower values for acetyl-CoA, indicating a higher affinity for this substrate than WT *Cp*GNA1. Regarding GlcN6P, all mutants displayed higher *K*_m_ values, suggesting a lower affinity for GlcN6P compared to WT. T117A showed the lowest *K*_m_ for acetyl-CoA (*K*_m_ = 33.41 ± 11.82 μM, which is a 15.9-fold *K*_m_ decrease compared to WT) and H81A the highest *K*_m_ for GlcN6P (*K*_m_ = 4,007 ± 1,425 μM, which is a 26-fold *K*_m_ increase compared to WT). Nevertheless, T117A and H81A showed remarkably low catalytic efficiencies for both acetyl-CoA and GlcN6P. Although it was expected to see a weak affinity for GlcN6P in the T117A mutant, the higher affinity for acetyl-CoA was not predicted because Thr117 shows only interactions with the pseudosubstrate Glc6P (Fig. 5). The relevance of key residues for substrate interaction, especially those related to GlcN6P binding (i.e., Arg73, Arg113, and Thr117), which are not conserved in *Hs*GNA1, strongly prompts further exploration of the enzyme as a selective drug target against P. falciparum and, possibly, other apicomplexan parasites. Overall, and except for Y127A, our results suggest that most of the mutants with alterations around the GlcN6P binding site show more significant effects on the kinetic properties under variable GlcN6P concentrations than the corresponding mutants show with acetyl-CoA. This implies a more significant role of the native residues around the GlcN6P binding site than the residues around acetyl-CoA.

## DISCUSSION

In this work we use CRISPR-Cas9-based engineering techniques (20), combined with a DiCre-recombinase system, for conditional P. falciparum *gna1* gene disruption upon rapamycin induction (19, 21, 35). This is a widely used and versatile strategy for rapid conditional gene ablation and functional analysis of essential genes in the malaria parasite. The data demonstrate that *Pf*GNA1, which acetylates GlcN6P in the amino sugar pathway, is required for the synthesis of adequate pools of UDP-GlcNAc and for parasite development during the asexual intraerythrocytic stages of the parasite. This confirms results from large-scale genetic screenings recently carried out in P. falciparum, underlining the relevance of the enzymes in the amino sugar pathway for the survival of asexual parasites (11). Remarkably, works performed in a P. berghei murine model of malaria also highlight the critical role of UDP-GlcNAc and the amino sugar metabolic route in mosquito and liver stages (12). However, contrasting to P. falciparum (11), P. berghei mutants in the UDP-GlcNAc biosynthetic route showed only slow-growth phenotypes in asexual intraerythrocytic parasites, indicating the nonessentiality of the pathway at these stages of the life cycle (12, 36). Despite that a *gna1* mutant has not been generated and UDP-GlcNAc levels have not been characterized in P. berghei (12, 36), this suggests that murine parasites can obtain UDP-GlcNAc from host cells or alternative sources along intraerythrocytic asexual development (12). Indeed, there exist other examples in which the alteration of a *de novo* sugar nucleotide metabolic route does not cause a significant reduction of the pathway’s product (37), and our own data show that P. falciparum *gna1* mutant growth is rescued *in vitro* by GlcNAc medium supplementation. Thus, P. falciparum parasites seem to be able to take up and activate GlcNAc when the amino sugar pathway is disrupted (38), in agreement with the wide range of sugars that can be imported through its hexose transporter 1 (39). Nevertheless, P. falciparum potential GlcNAc uptake is deemed to be negligible *in vivo*, as this is not an abundant free sugar in the parasite host (40), opening the door to explore the selective inhibition of *Pf*GNA1 as a new approach to treat malaria.

To identify structural differences between *Pf*GNA1 and *Hs*GNA1 that could be exploited for selective drug design, we intended to solve the P. falciparum enzyme 3D structure. However, the deficient expression of *Pf*GNA1 prompted us to explore *Cp*GNA1 as a surrogate model for Apicomplexa-specific GNA1 (10). Fortunately, *Cp*GNA1 is expressed at high levels in Escherichia coli, allowing us to obtain structural and enzymatic insights. Remarkably, despite the low identity at sequence level between *Cp*GNA1 and *Hs*GNA1, the crystal structures are similar and share the typical acetyltransferase fold (23). Although this had been already described in members of the GNAT superfamily, which show well-conserved structures and catalytic mechanisms despite extensive divergence at the sequence level (29, 32, 33), the independently evolved Apicomplexa GNA1 family still presents different catalytic residues crucial for enzymatic activity (10). Though both the *Cp*GNA1 acetyl-CoA and GlcN6P binding sites are different, some features are conserved, such as the highly conserved and critical tyrosine residue at position 127 (Tyr127), also present in nonapicomplexan GNA1 enzymes (29, 32, 33). At the level of the GlcN6P binding site, the main differences are localized in two arginine residues at positions 73 and 113, together with histidine, threonine, and glutamic acid residues at positions 81, 117, and 119, respectively. At the level of the acetyl-CoA binding site, the main differences are localized in histidine, asparagine, isoleucine, lysine, and leucine at positions 81, 90, 122, 125, and 126, respectively. Site-directed mutagenesis and kinetic assays confirmed the essential function of Tyr127. Likewise, GNA1 activity was not detected in our R141A mutant, suggesting that the positive charge of the arginine, which is also conserved in *Hs*GNA1, might interact with the negatively charged atoms on the phosphate group of GlcN6P or facilitate the interaction of Arg113 with the phosphate group. Furthermore, our analyses showed that Arg73 and Arg113 residues are relevant for GlcN6P affinity, whereas His81 is important for both GlcN6P and acetyl-CoA binding, in agreement with our structural data showing that His81 interacts with both ligands. Although our data reveal that Thr117 is important for GlcN6P and acetyl-CoA binding, the latest is not completely understood because this residue only interacts with the sugar moiety. Overall, there are remarkable differences between *Cp*GNA1 and *Hs*GNA1 at the level of GlcN6P and acetyl-CoA binding sites, which could be exploited for drug selectivity against parasites.

The amino sugar pathway has been explored in different organisms as a possible source of novel therapeutic targets, and inhibitors of the Aspergillus fumigatus GNA1 enzyme have been recently identified (32, 33, 41). The independent evolution and sequence divergence of apicomplexan GNA1, together with its essentiality in P. falciparum, highlight the potential of the enzyme as a selective therapeutic target against the malaria parasite (10). Furthermore, the recent identification of the amino sugar pathway as an important metabolic bottleneck in P. falciparum indicates that the inhibition of *Pf*GNA1 could largely hamper the growth of the parasite (9). Remarkably, UDP-GlcNAc has also been reported as the most limiting sugar nucleotide in mosquito and liver stages, and, as stated above, several enzymes in the metabolic route have been proven to be essential for parasite transmission to mosquitoes in a malaria murine model (12). Altogether, the data underline the potential of *Pf*GNA1, central to the amino sugar metabolic pathway, as a multistage drug target against P. falciparum. Interestingly, considering the particularities of GNA1 in the Apicomplexa and the importance of the UDP-GlcNAc donor for the biology of these organisms, the selective inhibition of the enzyme might also provide therapeutic opportunities against other apicomplexan pathogens such as C. parvum or Toxoplasma gondii (42, 43).

## MATERIALS AND METHODS

### P. falciparum culture and transfection.

P. falciparum asexual stages were cultured at 37°C in an atmosphere of 92% N_2_, 3% O_2_, and 5% CO_2_ with washed B+ blood type red blood cells (RBCs) at 2 to 4% hematocrit in Albumax-containing RPMI 1640 medium. Human erythrocytes were purchased from the Banc de Sang i Teixits (Catalonia, Spain), after approval from the Comitè Ètic Investigació Clínica Hospital Clínic de Barcelona. Parasite growth was monitored by counting the infected erythrocytes in Giemsa-stained blood smears by light microscopy. Parasites harboring a floxed region of *gna1* were generated by Cas9-mediated gene replacement using a pDC2 Cas9- and specific-single guide RNA (sgRNA)-expressing construct, and a linearized pUC19 plasmid containing the engineered *gna1* sequence (19). P. falciparum 3D7 II-3 parasites, with the DiCre system inserted into the *p230p* genomic locus (a generous gift from Ellen Knuepfer [19]), were transfected during ring stages, as described elsewhere (37).

Parasites recovered after transfection were harvested for genotyping. Genomic DNA from parasites was extracted with the QIAmp DNA minikit (Qiagen) following the manufacturer’s instructions. Purified samples were used as a template for PCR amplification of the inserted construct. Clonal parasite lines were then obtained from engineered populations by limiting dilution.

### P. falciparum growth analysis.

For rapamycin treatment experiments, 0.1% DMSO or 10 nM rapamycin was added to the culture medium in ring-stage-synchronized parasites, incubated for 1 h, and washed away (19). This was done by adding 1:1,000 (vol/vol) stocks of 100% DMSO or 10 μM rapamycin, respectively, to the culture medium. Genomic DNA was recovered 24 to 30 h later. For growth assessment after rapamycin treatment, 200 μl of parasite culture at 3% hematocrit and with an initial parasitemia of 0.1% were transferred to 96-well plates. Parasitemia was assessed by flow cytometry after 48 h, 96 h, and 144 h, using SYTO11 as previously described (44).

### Sugar nucleotide analysis.

Asexual blood stage parasite cultures were sorbitol-synchronized (or combining Percoll and sorbitol treatments, for tight synchronization) and osmotic lysis was performed on trophozoite-infected red cells at 35 to 40 h postinvasion (5 to 10% parasitemia). Sugar nucleotides were analyzed by liquid chromatography-tandem mass spectrometry (LC-MS/MS) and multiple-reaction monitoring (MRM) in a QTRAP 6500 System (AB Sciex). The metabolites, separated using a Hypercarb PGC column (5 μm, 2.1 × 100 mm; Thermo Fisher Scientific) with a mobile phase composed of 0.1% formic acid (pH 9) and an acetonitrile gradient (24), were identified by their diagnostic MRM transitions (38).

### Expression and purification of *Cp*GNA1.

The pGEX6P1*-CpGNA1* plasmid encoding the *Cp*GNA1 protein C terminus fused to the glutathione *S*-transferase (GST), with a PreScission Protease (PP) cleavage site (LEVLFQGP) encoded in the linker between GST and *Cp*GNA1, was transformed into the Escherichia coli expression strain BL21 Star (Thermo Fisher Scientific). Transformants were selected on LB (Sigma-Aldrich) plates containing 100 μg/ml of ampicillin. Batch cultures were scaled up to 2 liters of LB medium, and cells were grown to an optical density of 0.5 to 0.6 at 37°C and 180 rpm. They were subsequently induced with 1 mM isopropyl-β-d-thiogalactopyranoside (IPTG) (Sigma-Aldrich). After 20 h of induction at 18°C, the cells were collected by centrifugation at 11,295 × *g* for 20 min. The resulting pellet was suspended in 60 ml of binding buffer A (25 mM TRIS [pH 7.5], 150 mM NaCl) that was supplemented with 4 μl of Universal Nuclease for Cell Lysis (Thermo Fisher Scientific, 250 U/μl), lysozyme (Sigma-Aldrich, >95% pure) up to a final concentration of 1 mg/ml, and 500 μl of a protease-inhibitor cocktail solution (1 M PMSF, 10 mM benzamidine, 0.5 mM leupeptine). This solution was incubated for 30 min at 37°C and then sonicated in ice for 15 cycles, each consisting of a 30-s pulse plus a 30-s cooling step. The lysate was afterward centrifuged at 39,086 × *g* for 20 min.

The supernatant was filtered through a 0.45-μm membrane and loaded at 1 ml/min into two connected GSTrap 4B (GE Healthcare) columns, previously equilibrated with buffer A. The columns were washed with buffer A until no 280-nm absorbance was observed, and the bound protein was eluted by a 25-min gradient from buffer A to buffer B (25 mM TRIS, 150 mM NaCl, 400 mM l-glutathione reduced [Sigma-Aldrich] [pH 7.5]). The recombinant fusion protein containing *Cp*GNA1 was then exchanged into buffer C (25 mM Tris [pH 7.5], 150 mM NaCl) and incubated overnight at 4°C with PP at a ratio of 1 mg of PP per 50 mg of fusion protein, in order to remove the GST protein, leaving the GPLGS amino acids at the *Cp*GNA1 N terminus. Once the cleavage was verified by SDS-PAGE, the mixture was loaded into three coupled GSTrap 4B columns, previously equilibrated in buffer A. Both GST and PP remained fixed to the column matrices, and the *Cp*GNA1 was collected as the unbinding fraction. *Cp*GNA1 was finally purified by size exclusion chromatography using a HiLoad 26/60 Superdex 75 Column (GE Healthcare), previously equilibrated with buffer C. Fractions containing *Cp*GNA1 were dialyzed against buffer D (25 mM Tris pH 7.5), concentrated, and used for biophysical experiments. Quantification of the enzyme was carried out by absorbance at 280 nm using its theoretical extinction coefficient. A yield of 15 mg/liter of culture was obtained.

### *Cp*GNA1 crystallization, phasing, and refinement.

Crystal forms for *Cp*GNA1 were obtained when CpGNA1 (1.5 mg/ml) was cocrystallized with 10 mM acetyl-CoA. The sitting-drop vapor-diffusion method was used to produce crystals by mixing 0.5 μl of the protein-ligand solution with an equal volume of mother liquor (0.2 M NH_4_Cl, 0.1 M HEPES [pH 7.5], 25% glycerolethoxilate) at 18°C. Bar-like-shaped crystals grew within 1 day. The crystals were soaked for 45 min with a mixture containing 50 mM acetyl-CoA and 50 mM Glc6P in the mother liquor described above. Then, crystals that were either cocrystallized with acetyl-CoA or further soaked with acetyl-CoA and Glc6P were cryoprotected using 25% glycerol in the mother liquor and frozen in nitrogen gas stream cooled to 100 K. X-ray diffraction data were collected at XALOC-13 beamline at ALBA Synchrotron. Data were processed and scaled using XDS (45) and CCP4 (46, 47) software packages. Relevant statistics are given in Table S1 in the supplemental material.

A promising partial solution for the monomeric *Cp*GNA1 was identified for the P2_1_2_1_2 crystal form (crystals cocrystallized with acetyl-CoA) from a data set diffracting anisotropically to 1.5 Å, using the multisolution parallel phasing software ARCIMBOLDO_LITE (48). ARCIMBOLDO uses PHASER to place individual α-helices by eLLG-guided molecular replacement (49) and then expand partial solutions with SHELXE (50) through density modification and autotracing. The search was set to locate 1 copy of an ensemble generated with an alpha version of the software ALEPH (51) The ensemble of two models represented the probable fold composed by 5 beta strands flanked by 2 alpha helices at each side and it contained the GNA1 of Saccharomyces cerevisiae (PDB id 1I21, 19% identity to target sequence) and the acetyltransferase from Agrobacterium tumefaciens (PDB 2DXQ, 26% identity to target sequence). The ARCIMBOLDO_LITE solution was completed by SEQUENCE SLIDER (26) using side chain modeling with Scwrl4 (52), refinement with REFMAC5 (53), and expansion with SHELXE, resulting in a best trace of 120 residues with CC 31.9. The structure of *Cp*GNA1 with the substrates at 1.95 Å, in space group P2_1_2_1_2_1_, containing two monomers in the asymmetric unit was phased by molecular replacement (27) using the previous model. Initial phases were further improved by cycles of manual model building in Coot (54) and refinement with REFMAC5 (53) and Phenix (55). Further rounds of Coot and refinement with Phenix (55) were performed to obtain the final structure. The final model was validated with PROCHECK, and model statistics are given in Table S1. The Ramachandran plots for the *CpGNA1*-acetyl-CoA-Glc6P show that 89.0%, 10.6%, 0.4%, and 0% of the amino acids are in most favored, allowed, generously allowed, and disallowed regions, respectively. PyMOL (56) was used to generate images.

### *Cp*GNA1 colorimetric *in vitro* enzyme kinetic assays.

Codon-optimized versions of *Cp*GNA1 WT and site-specific mutant proteins were expressed and purified as previously described (10). Briefly, sequences were cloned in a pGEX 6P-1 vector containing an N-terminal GST-tag. After induction, fusion proteins were purified by affinity chromatography and eluted with reduced glutathione without cleaving the GST-tag. *In vitro* activity colorimetric assays were based on the detection of coenzyme A, generated during acetyl transfer, by reaction with the thiol reagent 5,5′-dithiobis(2-nitrobenzoic acid) (DTNB). Acetyl-CoA and GlcN-6P were used as the substrates and all the reactions were carried out at room temperature, in triplicate, in a 96-well-plate format. *Cp*GNA1 was assayed in buffer solution (25 mM Tris-HCl and 150 mM NaCl, pH 7.2) using different acetyl-CoA concentrations and a fixed GlcN-6P concentration (2,000 μM), or different GlcN-6P concentrations and a fixed acetyl-CoA concentration (500 μM). *Cp*GNA1 WT was assayed at a 5 μg/ml concentration whereas *Cp*GNA1 mutants were tested at a 25 μg/ml concentration. The data were fitted to a nonlinear least-squares regression using GraphPad Prism.

10.1128/mBio.02045-20.1 FIG S1 Giemsa-stained representative microscopy images (1,000× magnification) of *gna1-loxP* parasites. (A) DMSO-incubated parasite *gna1-loxP* culture at IDC 2. (B) Rapamycin-incubated parasite *gna1-loxP* culture at IDC 2. Black arrowheads indicate early forms (rings), grey arrowheads show merozoites, and green arrowheads mark mature forms (trophozoites and schizonts). Download FIG S1, PDF file, 0.5 MB. Copyright © 2020 Chi et al. 2020 Chi et al. This content is distributed under the terms of the Creative Commons Attribution 4.0 International license.

10.1128/mBio.02045-20.2 FIG S2 Polder mFobs. DFmodel maps at 3.0 (A) and 2.5 sigma (B) show clear electron density for most of the atoms of the AcCoA and G6P. The ligands are colored as green carbon atoms. Download FIG S2, PDF file, 2.0 MB. Copyright © 2020 Chi et al. 2020 Chi et al. This content is distributed under the terms of the Creative Commons Attribution 4.0 International license.

10.1128/mBio.02045-20.3 FIG S3 Sequence alignment of *Cp*GNA1 and *Hs*GNA1. Black labeled residues indicate identity/high similarity. Note that for both enzymes, only the residues present in the crystal structures are shown in the alignment. Shown above the sequences for *Cp*GNA1 and *Hs*GNA1, in red and green, and in blue and magenta, are displayed the secondary structure elements (α-helices and β-strands, respectively). Download FIG S3, PDF file, 1.0 MB. Copyright © 2020 Chi et al. 2020 Chi et al. This content is distributed under the terms of the Creative Commons Attribution 4.0 International license.

10.1128/mBio.02045-20.4 Table S1 Data collection and refinement statistics of *Cp*GNA1-acetyl-CoA and *Cp*GNA1-acetyl-CoA-Glc6P complexes. Values in parentheses refer to the highest resolution shell. Ramachandran plot statistics were determined with PROCHECK. Download Table S1, DOCX file, 0.02 MB. Copyright © 2020 Chi et al. 2020 Chi et al. This content is distributed under the terms of the Creative Commons Attribution 4.0 International license.
