# Supplementary figures and images for: Plasmodium falciparum Apicomplexan-Specific Glucosamine-6-Phosphate N-Acetyltransferase Is Key for Amino Sugar Metabolism and Asexual Blood Stage Development
Source: mBio. 2020 Oct 20;11(5):e02045-20. doi: 10.1128/mBio.02045-20 (PMC7587441; doi:10.1128/mBio.02045-20)

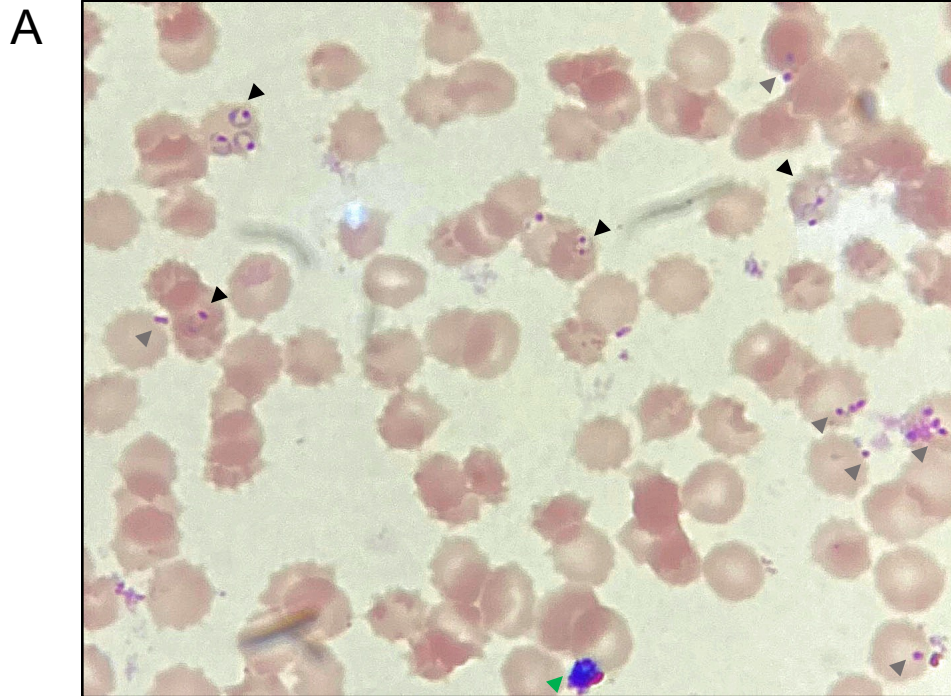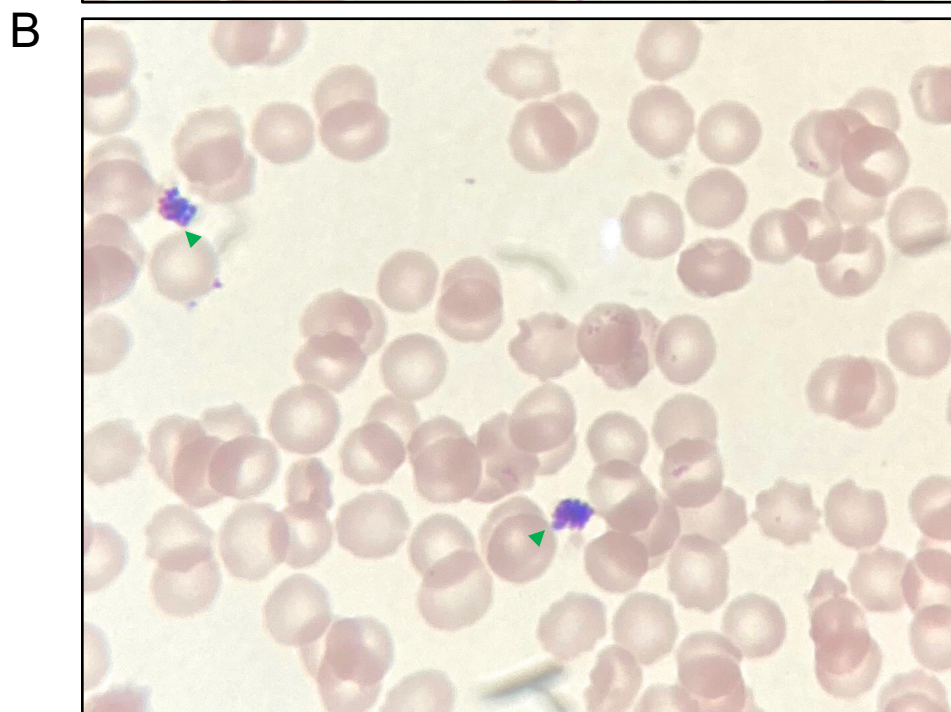

Supplement: FIG S1 [file mBio.02045-20-sf001.pdf]

A

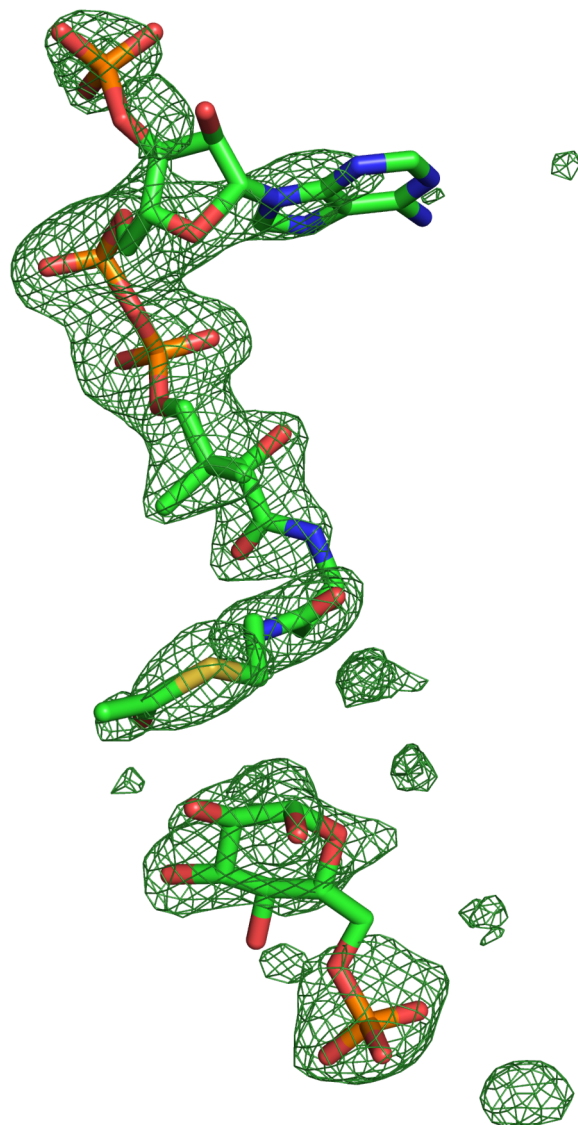

B

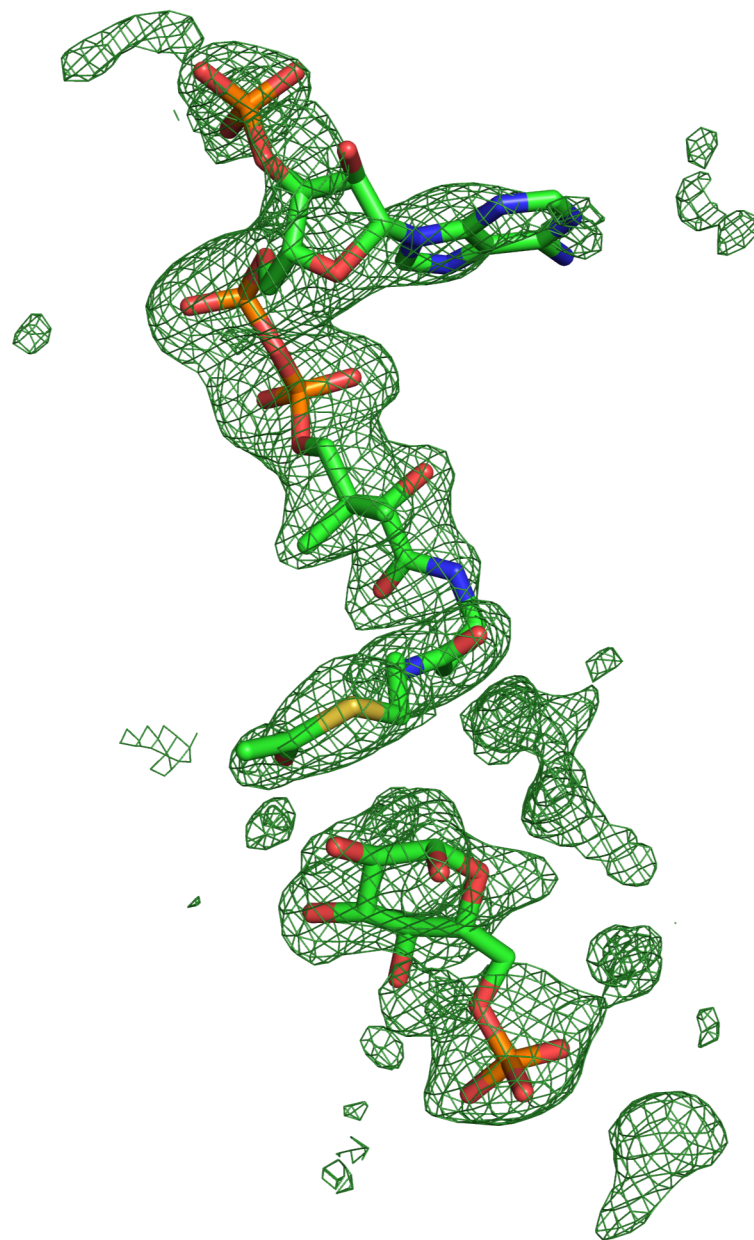

Supplement: FIG S2 [file mBio.02045-20-sf002.pdf]

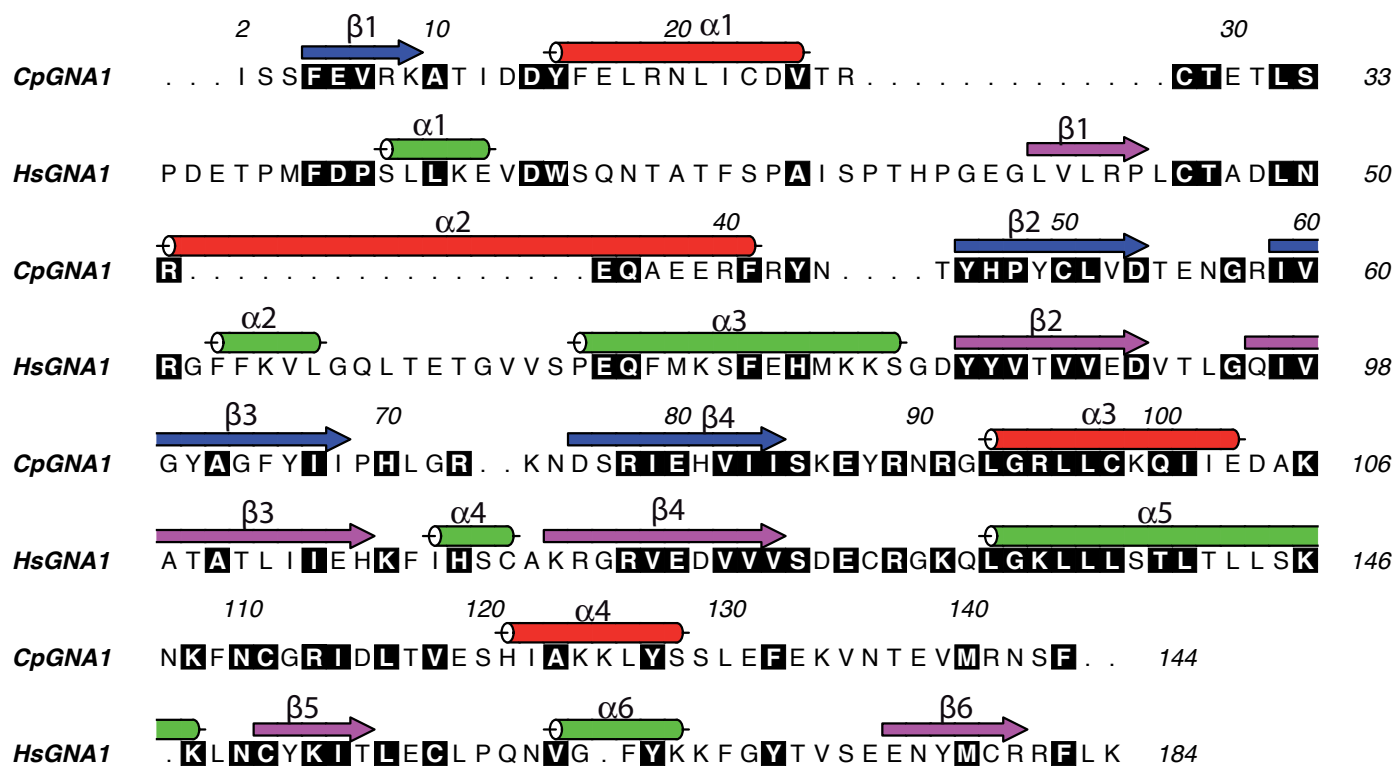

Supplement: FIG S3 [file mBio.02045-20-sf003.pdf]
