# Supplementary material for: Plasmodium falciparum Apicomplexan-Specific Glucosamine-6-Phosphate N-Acetyltransferase Is Key for Amino Sugar Metabolism and Asexual Blood Stage Development
Source: mBio. 2020 Oct 20;11(5):e02045-20. doi: 10.1128/mBio.02045-20 (PMC7587441; doi:10.1128/mBio.02045-20)
Supplement: Table S1 [file mBio.02045-20-st001.docx]

**Supplementary Table I.** Data collection and refinement statistics of complex ***Cp*GNA1-acetyl-CoA-Glc6P**. Values in parentheses refer to the highest resolution shell. Ramachandran plot statistics were determined with PROCHECK.

|  | ***Cp*GNA1-acetyl-CoA** | ***Cp*GNA1-acetyl-CoA-Glc6P** |
| --- | --- | --- |
| Space group | P2_1_2_1_2 | P2_1_2_1_2_1_ |
| Wavelength (Å) | 0.97 | 0.97 |
| Resolution (Å) | 20.00-1.50  (1.58-1.50) | 20.00-1.95  (2.06-1.95) |
| Cell dimensions (Å) | a = 55.67  b = 72.18  c = 35.03 | a = 55.69  b = 70.09  c = 71.85 |
| Unique reflections | 22382 | 20192 |
| Completeness | 96.3 (97.1) | 95.9 (87.7) |
| *R*_pim_ | 0.030 (0.892) | 0.056 (0.555) |
| Mn(I) half-set correlation CC(1/2*)* | 0.999 (0.608) | 0.998 (0.852) |
| *I*/σ(*I*) | 10 (1.0) | 6.9 (1.1) |
| Redundancy | 96.3 (97.1) | 4.7 (3.3) |
| *R*_work_ / *R*_free_ |  | 0.272/0.313 |
| RMSD from ideal geometry, bonds (Å) |  | 0.009 |
| RMSD from ideal geometry, angles (º) |  | 1.353 |
| <*B*> protein (Å^2^) |  | 53.99 |
| <*B*> acetyl-CoA  (Å^2^) |  | 71.2 |
| <*B*> Glc6P  (Å^2^) |  | 81.9 |
| <*B*> solvent (Å^2^) |  | 52.10 |
| PDB ID |  | 6YUG |
